# The mediating role of employees engagement in the relationship between adaptive leadership and service quality in the health sector

**DOI:** 10.1108/LHS-03-2025-0056

**Published:** 2025-06-10

**Authors:** Biniam Ali Eshete, Tilaye Kassahun

**Affiliations:** DBL, University of South Africa, Pretoria, South Africa; Department of Business Management, Ethiopian Civil Service University, Addis Ababa, Ethiopia

**Keywords:** Adaptive leadership, Employee engagement, Service quality, Health care, Mediation effect

## Abstract

**Purpose:**

This study aims to examine the mediating role of employee engagement in the relationship between adaptive leadership and service quality within the health-care sector in Ethiopia.

**Design/methodology/approach:**

A quantitative research design was adopted, using a cross-sectional survey method. Data was collected from health-care professionals through structured questionnaires. Partial least squares structural equation modeling was used to analyze the hypothesized relationships.

**Findings:**

This study found that adaptive leadership (ADL) indirectly enhances service quality (SVQ) through employee engagement (EME). While ADL had no direct effect on SVQ, it positively influenced EME, which, in turn, significantly improved SVQ. These findings emphasize the critical role of employee engagement in translating leadership into better service outcomes in health-care setting.

**Practical implications:**

The findings offer valuable insights for health-care managers and policymakers, emphasizing the need for ADL strategies that foster employee engagement to improve SVQ.

**Originality/value:**

This study uniquely examines how ADL translates into improved health-care SVQ through the engagement of frontline staff in a dynamic and resource-constrained environment.

## 1. Introduction

The health-care landscape today is characterized by unprecedented challenges. The lingering effects of the COVID-19 pandemic, coupled with rapid technological advancements, evolving patient expectations and persistent resource constraints, demand leadership that can navigate ambiguity and foster resilience (Heifetz *et al.*, 2019). In this dynamic environment, adaptive leadership emerges as a crucial approach. Rooted in the understanding that complex problems often lack straightforward solutions, adaptive leadership is defined by the ability of leaders to diagnose these challenges, encourage experimentation and learning across the organization and empower individuals at all levels to tackle them collaboratively (Northouse, 2019). This involves creating a supportive context where employees feel psychologically safe to take risks, challenge existing norms and contribute innovative solutions, ultimately fostering organizational agility.

A critical element in translating adaptive leadership into positive organizational outcomes is employee engagement. Going beyond mere job satisfaction, employee engagement refers to the emotional, cognitive and behavioral investment that employees have in their work and their organization (Schaufeli, 2021). Engaged employees exhibit a strong sense of connection to their work, are enthusiastic about their roles and are willing to go the extra mile to contribute to organizational success. This heightened level of dedication translates into greater productivity, lower turnover rates and a more positive work environment (Kaya and Karatepe, 2020). Crucially, engaged employees are more likely to be attentive to patient needs, demonstrate empathy and contribute to a positive service climate, directly impacting the quality of care delivered.

Ultimately, the effectiveness of health-care organizations is fundamentally judged by the service quality they provide. This encompasses the totality of the patient experience, extending beyond clinical outcomes to include the responsiveness of staff, the clarity of communication, the empathy demonstrated, the efficiency of processes and the overall environment of care (Restivo *et al.*, 2022). High service quality is not only essential for patient satisfaction and trust, which are increasingly linked to organizational reputation and financial performance but also plays a vital role in fostering patient loyalty and positive word-of-mouth referrals.

While the individual importance of adaptive leadership, employee engagement and service quality in health care has been acknowledged in the literature (Northouse, 2019; Restivo *et al.*, 2022; W. Schaufeli, 2021), a significant gap remains in understanding the precise interplay between these constructs, particularly the mechanisms through which adaptive leadership influences service quality. Existing research has predominantly focused on other leadership paradigms, such as transformational or transactional leadership, in relation to employee engagement and service quality (Specchia *et al.*, 2021; Yukl and Mahsud, 2010). Even though the relationship between leadership, employee engagement and service quality has been extensively examined, most studies are concentrated in developed economies, where institutional, cultural and organizational contexts differ significantly from those in developing countries (Pahi *et al.*, 2020). This geographical and contextual concentration limits the applicability of prevailing theories, as leadership is not only a function of individual behavior, but also deeply rooted in the context of the environment (Avolio *et al.*, 2009; Brauckmann *et al.*, 2023). The Ethiopian health sector is an example of a setting where traditional management models may not be easily applied due to continuing resource constraints, hierarchical organizational structures and ongoing systemic reforms (Boku *et al.*, 2024; Teame *et al.*, 2022). These conditions underline the need for empirical research explicitly addressing contextual complexity, although adaptive leadership has been proposed as a particularly appropriate tool for managing uncertainty and change (Ronald Heifetz, 2009; Uhl-Bien and Arena, 2018). Moreover, existing literature largely emphasizes direct effects, often overlooking the processes and conditions through which leadership produces outcomes particularly the mediating role of employee engagement. Addressing this gap is crucial for advancing leadership theory and offering practical insights for underrepresented sectors and regions. This study responds by examining how adaptive leadership shapes service quality in Ethiopia’s private health-care sector, with a focus on employee engagement as a key facilitating mechanism in resource-constrained, culturally distinct settings. Specifically, the study addresses the following research questions:

*RQ1*. How does adaptive leadership influence service quality in private hospitals in Ethiopia?

*RQ2*. What is the effect of adaptive leadership on employee engagement in this context?

*RQ3*. How does employee engagement affect service quality?

*RQ4*. Does employee engagement mediate the relationship between adaptive leadership and service quality?

## 2. Literature review and hypothesis development

### 2.1 Theoretical foundation

This study is anchored in adaptive leadership theory (Heifetz *et al.*, 2009), which posits that effective leadership in complex and dynamic environments necessitates the ability to mobilize individuals to tackle challenging problems through learning, adaptation and collaborative problem-solving, rather than relying solely on hierarchical authority or predefined solutions. In the context of health care, characterized by constant flux in patient needs, technological advancements and regulatory landscapes, adaptive leadership provides a crucial framework for fostering organizational agility and resilience (Karreinen *et al.*, 2023). It emphasizes the leader’s role in diagnosing adaptive challenges, creating a holding environment for difficult work and empowering stakeholders to develop and implement solutions.

Complementing this theoretical lens is the job demands-resources (JD-R) model (Bakker and Demerouti, 2017). The JD-R model posits that employee well-being and performance are a result of the balance between job demands (aspects of the job that require sustained physical or mental effort) and job resources (aspects of the job that are functional in achieving work goals, reducing job demands and stimulating personal growth). In this study, we conceptualize adaptive leadership as a key job resource. Leaders who exhibit adaptive behaviors provide support, foster autonomy and promote learning, thereby mitigating the impact of high job demands prevalent in health care and fostering employee engagement, which in turn drives service quality. The integration of these two theories allows for a comprehensive examination of how adaptive leadership not only directly influences service quality but also indirectly enhances it by bolstering employee engagement as a vital resource.

### 2.2 Adaptive leadership: a distinct approach in a digital era

In today’s digital era, adaptive leadership stands out as a crucial approach for navigating rapid technological transformations. Malik *et al.* (2024) highlighted how traditional leadership styles no longer suffice in the face of dynamic digital changes; leaders must now exhibit agility, openness and innovative thinking to successfully drive digital transformation. Similarly, Zia *et al.* (2024) argued that digital leadership deeply intertwined with adaptive qualities enables organizations to foster digital engagement among employees, cultivate digital leadership capabilities internally, and, ultimately, promote innovative work behavior. Adaptive leaders in this era are distinguished by their ability to embrace continuous learning, data-driven decision-making and a customer-centric mindset, blending traditional leadership strengths with the new demands of a digitally connected world.

Moreover, the research by Faiz *et al.* (2024) underscores that mastering digital leadership capabilities is not just about adopting new technologies but about reinventing business models to sustain competitiveness. Adaptive leaders facilitate this by guiding managerial decision-making processes that align digital strategies with organizational goals, often leveraging resources like innovation grants to support transformation efforts. This distinct leadership approach is pivotal in helping organizations remain flexible, customer-focused and innovation-driven amidst the volatility of digital markets. Therefore, adaptive leadership in the digital era is not merely an evolution of style but a fundamental shift toward resilience, innovation and strategic agility (AlNuaimi *et al.*, 2022; Huang *et al.*, 2023).

### 2.3 Adaptive leadership and service quality

Adaptive leadership emphasizes a leader’s capacity to encourage learning, adaptability and collaborative problem-solving in response to dynamic environments (Heifetz *et al.*, 2009). In health care, where responsiveness to rapid changes and patient-centric care are paramount, adaptive leadership is crucial for ensuring high service quality (Rojko *et al.*, 2025). By fostering a culture of continuous improvement and empowering frontline staff to address evolving patient needs, adaptive leaders can enhance patient satisfaction, improve service delivery efficiency and promote adherence to best practices (Hawley, 2021). Research indicates that health-care organizations with adaptive leadership practices tend to exhibit higher patient satisfaction scores and improved operational efficiency (Kaya and Karatepe, 2020).

Based on this, we hypothesize:

*H1*. Adaptive leadership has a positive effect on service quality in health-care organizations.

### 2.4 Adaptive leadership and employee engagement

Employee engagement, characterized by employees’ emotional commitment and willingness to exert effort (Gede and Huluka, 2024), is significantly influenced by leadership styles. Adaptive leaders foster a supportive and participative work environment by encouraging employees to embrace change, contribute ideas and take ownership (Uhl-Bien and Arena, 2018). This approach cultivates psychological safety, autonomy and a sense of meaningful work, all of which are strong predictors of employee engagement (W. Schaufeli, 2021). Empirical studies have shown a positive correlation between adaptive leadership and higher levels of staff engagement, reduced turnover and greater job satisfaction in health-care settings (Curado and Santos, 2022).

Therefore, we propose the following hypothesis:

*H2*. Adaptive leadership positively influences employee engagement in health-care organizations.

### 2.5 Employee engagement and service quality

A strong link exists between employee engagement and the quality of service delivered, particularly in service-oriented industries like health care (Harter *et al.*, 2002). Engaged employees are more likely to be proactive, empathetic and focused on delivering positive patient experiences (Bakker and Albrecht, 2018). Research consistently demonstrates that higher levels of employee engagement are associated with improved service quality metrics, including patient satisfaction and care quality (Boyle *et al.*, 2023; Schaufeli, 2021). Furthermore, engaged employees exhibit greater resilience and are better equipped to handle the demanding nature of health-care work, reducing the likelihood of burnout and maintaining a high level of service (Gou *et al.*, 2021).

Drawing on this evidence, we hypothesize:

*H3*. Employee engagement has a positive effect on service quality in health-care organizations.

### 2.6 The mediating role of employee engagement

We argue that the positive impact of adaptive leadership on service quality is not solely direct but is also mediated through employee engagement. Adaptive leaders create a work environment that fosters engagement by providing support, promoting autonomy and encouraging growth (Coleman, 2021). This engaged workforce is then more motivated and equipped to deliver high-quality service. The JD-R model supports this by suggesting that leadership acts as a job resource that enhances engagement, ultimately leading to positive outcomes like improved service quality (Baker *et al.*, 2020; Katou *et al.*, 2021; Miner and Bickerton, 2020). Empirical studies have indicated that employee engagement mediates the relationship between leadership styles (including adaptive leadership) and service outcomes in health care (Curado and Santos, 2022).

Based on this theoretical rationale and empirical evidence, we propose the following hypothesis:

*H4*. Employee engagement mediates the positive relationship between adaptive leadership and service quality in health-care organizations.

**Figure 1.**
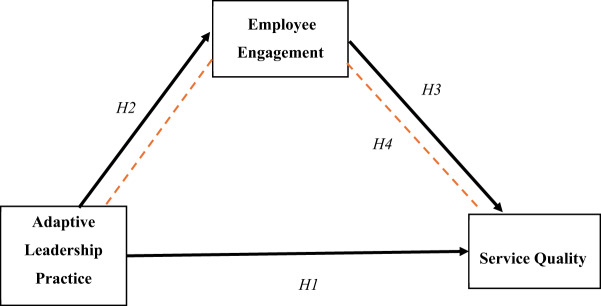
Conceptual framework of the study **Source:** Authors’ own work

Generally, the hypothesized relationships discussed above are illustrated in Figure 1, which presents the conceptual framework of the study.

## 3. Methodology

This study adopted a pragmatist research philosophy, prioritizing the research question and using quantitative methods to rigorously investigate the relationships between adaptive leadership, employee engagement and service quality within a unique and significant context: privately owned hospitals in Ethiopia. A descriptive and explanatory sequential research design was used, commencing with quantitative data collection and analysis to identify patterns and relationships, followed by interpretation and explanation of these findings in light of the specific context.

### 3.1 Study setting

The Ethiopian health-care sector presents a compelling and under-researched context for studying the impact of leadership on employee engagement and service quality (Boku *et al.*, 2024). As a rapidly developing nation, Ethiopia faces significant health-care challenges, including increasing demand for quality care, resource limitations and a dynamic health-care landscape characterized by a mix of public and private providers (Hundie and Habtewold, 2024). Private hospitals play a crucial and growing role in meeting the health-care needs of the population, often catering to a significant segment seeking potentially higher service standards. Understanding the leadership practices that foster employee engagement and drive service quality within this specific setting is vital for improving health-care delivery in Ethiopia and offers valuable insights for other developing nations with similar health-care system dynamics. This study, therefore, addresses a gap in the existing literature by providing empirical evidence from an African health-care context.

### 3.2 Sampling method and sample characteristics

A two-stage stratified random sampling technique was adopted to ensure a representative sample of both employees and the health-care institutions themselves. The target population for this study included all privately owned hospitals operating in Addis Ababa, Ethiopia. According to data from the Addis Ababa City Administration Health Bureau, a total of 41 privately owned hospitals were officially registered at the time of data collection.

In the first stage, 15 hospitals representing approximately 36.6% of the total population were selected using simple random sampling. In the second stage, participants were selected from one employee. A stratified random sampling method was used, with strata defined based on the size of the employee population in each of the 15 selected hospitals. This ensured proportional representation of employees from hospitals of varying sizes. A total of 347 employees were selected through this process. The unit of analysis for this research was the individual employee within the sampled hospitals.

### 3.3 Data collection process

Ethical approval for the study was obtained from the University of South Africa prior to data collection. Access to the selected hospitals and permission to conduct the survey were granted by the administrative heads of the 15 private hospitals in Addis Ababa. Data collection followed a structured process. A pretest was conducted using a sample of 30 employees from two private hospitals not included in the final study. This pretesting phase assessed the clarity, relevance and comprehensibility of the questionnaire, helping to identify and correct any ambiguities. Feedback from the pretest informed revisions to enhance the validity and reliability of the final instrument.

Trained research assistants administered the questionnaires in person at the selected hospitals. With supervisory approval, employee participants completed the surveys during working hours to minimize disruptions. Participants received a clear explanation of the study, were assured of confidentiality and anonymity, and signed informed consent forms before participating. Questionnaires were collected immediately after completion to ensure a high response rate. Data collection was conducted over six weeks, from October 10 to November 21, 2024. Upon completion, all questionnaires were reviewed for completeness and accuracy before data entry into SPSS for analysis.

### 3.4 Measurement scales

The study used established and validated measurement scales, adapted to the Ethiopian health-care context, to assess the key constructs. Adaptive leadership was measured using a scale developed by Nöthel *et al.* (2023), which captures various dimensions of adaptive leadership behavior as perceived by employees. Responses were recorded on a five-point Likert scale ranging from 1 (strongly disagree) to 5 (strongly agree). Employee engagement was assessed using the nine-item Utrecht Work Engagement Scale (UWES-9) by Schaufeli *et al.* (2006), covering the dimensions of vigor, dedication and absorption. Responses were also captured using a five-point Likert scale. Service quality was measured using an adapted version of the SERVQUAL scale (Parasuraman *et al.*, 1988), focusing on five dimensions relevant to health care: tangibles, reliability, responsiveness, assurance and empathy. The scale was refined based on feedback from the pretesting phase to suit the context of private hospitals in Ethiopia.

Reliability analysis using Cronbach’s alpha demonstrated acceptable internal consistency for all scales: 0.70 for adaptive leadership, 0.75 for employee engagement and 0.90 for service quality, all meeting or exceeding the recommended threshold of 0.70 (Tavakol and Dennick, 2011).

### 3.5 Method of data analysis

Data analysis involved both descriptive and inferential statistical techniques. Descriptive statistics were used to summarize respondents’ demographic characteristics and the overall levels of adaptive leadership, employee engagement and service quality. Inferential analysis was conducted using structural equation modeling (SEM) in Smart PLS version 4 to test the hypothesized relationships among the constructs and to examine the mediating role of employee engagement. SEM was chosen for its ability to simultaneously assess complex relationships among multiple variables. Model fit was evaluated using appropriate fit indices, while path coefficients were analyzed to determine the strength and direction of the relationships. Bootstrapping procedures were applied to assess the significance of the indirect effect of adaptive leadership on service quality through employee engagement.

## 4. Result

In the current study, we evaluated the structural model’s pathways using the partial least squares-structural equation modeling (PLS-SEM) method and the statistical software Smart PLS 4. PLS-SEM is a more appropriate method compared to other statistical methods, especially analyzing correlations between newly introduced variables (Schuberth *et al.*, 2023). In addition, we used Harman’s one-factor test to account for the possibility of common method bias resulting from the cross-sectional design of this study (Podsakoff *et al.*, 2012). The result indicates that the first component has an eigenvalue of 6.575, explaining 11.336% of the total variance. This is the highest value among all components, but it is still below 50%, indicating that no single factor dominates the data, which is a positive sign in terms of common method bias (Podsakoff *et al.*, 2003). As indicated in Table 1, the study surveyed 314 respondents, with a nearly balanced gender distribution (47.5% male and 52.5% female). Most participants were married (67.2%), while 29.3% were single, and a small proportion were divorced (2.5%) or preferred not to disclose their status (1.0%). In terms of education, the majority held a DSc/BA (56.7%), followed by MSc/MA (16.2%), college diploma (14.3%) and others (12.7%). The age distribution showed that 42.7% were between 31 and 40 years, 33.4% were 18–30 years, 14.0% were 41–50 years and 9.9% were above 51 years. Regarding work experience, the largest group had four to six years of experience (28.7%), while others had more than 10 years (25.5%), one to three years (20.7%), 7–10 years (20.1%) and less than one year (5.1%). The highest number of respondents worked in inpatient care (32.8%), followed by OPD (17.5%), pharmacy (15.9%), laboratory (11.1%), MCH (11.5%), administration (4.1%) and other divisions (7.1%).

**Table 1.** Respondents’ profile

Particulars	Response category	Frequency	%
Gender	Male	149	47.5
	Female	165	52.5
Marital status	Single	92	29.3
	Married	211	67.2
	Divorced	8	2.5
	Preferred not to say	3	1.0
Education level	College diploma	45	14.3
	DSc/BA	178	56.7
	MSc/MA	51	16.2
	Others	40	12.7
Age	18–30 years	105	33.4
	31−40 years	134	42.7
	41−50 years	44	14.0
	> 51 years	31	9.9
Experience	Less than 1 year	16	5.1
	1–3 years	65	20.7
	4–6 years	90	28.7
	7–10 years	63	20.1
	>10 years	80	25.5
Work division	Laboratory	35	11.5
	Pharmacy	50	15.9
	OPD	55	17.5
	MCH	36	11.5
	Inpatient	103	32.8
	Administration	13	4.1
	Other	22	7.1
*N* = 314			

**Source(s):** Authors’ own work

### 4.1 Measurement model evaluation

The measurement model was tested according to the criteria of internal consistency, convergent validity and discriminant validity. Measurement model evaluation as indicated in Table 2 demonstrates strong reliability and validity across constructs. Cronbach’s alpha and composite reliability (CR) values for all dimensions exceed the 0.7 threshold, confirming internal consistency (Hair *et al.*, 2019). Average variance extracted (AVE) values are above 0.5, indicating good convergent validity (Fornell and Larcker, 1981). Factor loadings range from 0.658 to 0.926, further supporting construct validity. Discriminant validity is established using the Fornell−Larcker criterion (Table 3) and the HTMT test (Table 4), with AVE square roots higher than interconstruct correlations and HTMT values below 0.9 (Henseler *et al.*, 2015). These results confirm that the measurement model effectively captures the constructs, ensuring robust structural model evaluation (Table 5).

**Table 2.** Measurement model result

Construct	Dimensions	Items code	Loading	Cronbach’s alpha	CR	AVE
Employee engagement	Absorption	AB1	0.823	0.815	0.820	0.731
		AB2	0.844			
		AB3	0.896			
	Vigor	V1	0.829	0.836	0.846	0.753
		V2	0.891			
		V3	0.881			
	Dedication	D1	0.876	0.699	0.730	0.629
		D2	0.658			
		D3	0.829			
Adaptive leadership	Give the way back to people	GWB1	0.794	0.828	0.829	0.660
		GWB2	0.802			
		GWB3	0.82			
		GWB4	0.833			
	Identify the adaptive challenge	IAC2	0.9	0.823	0.851	0.742
		IAC3	0.926			
		IAC4	0.747			
	Maintain disciplined attention	MDA1	0.848	0.824	0.872	0.654
		MDA2	0.872			
		MDA3	0.845			
		MDA4	0.651			
	Protect leadership voice below	PLV1	0.884	0.849	0.852	0.692
		PLV2	0.754			
		PLV3	0.801			
		PLV4	0.881			
	Regulate distress	RD1	0.81	0.851	0.920	0.685
		RD2	0.905			
		RD3	0.798			
		RD4	0.792			
Service quality	Responsiveness	RES1	0.772	0.810	0.828	0.634
		RES2	0.745			
		RES3	0.834			
		RES4	0.831			
	Reliability	REL1	0.845	0.861	0.861	0.705
		REL2	0.852			
		REL3	0.84			
		REL4	0.822			
	Tangibility	T1	0.795	0.876	0.882	0.730
		T2	0.886			
		T3	0.919			
		T4	0.813			
	Assurance	AS1	0.842	0.843	0.889	0.674
		AS2	0.724			
		AS3	0.87			
		AS4	0.841			
	Empathy	EE1	0.84	0.862	0.866	0.709
		EE2	0.868			
		EE3	0.893			
		EE4	0.761			

**Source(s):** Authors’ own work

**Table 3.** Discriminant validity Heterotrait-monotrait test

	AS	AB	D	EE	GWB	IAC	MDA	PLV	RD	RES	REL	T	V
AS													
AB	0.132												
D	0.063	0.298											
EE	0.152	0.270	0.185										
GWB	0.088	0.242	0.280	0.119									
IAC	0.062	0.240	0.204	0.242	0.204								
MDA	0.070	0.079	0.092	0.195	0.120	0.056							
PLV	0.051	0.151	0.242	0.229	0.475	0.165	0.175						
RD	0.060	0.148	0.235	0.049	0.122	0.095	0.149	0.070					
RES	0.100	0.122	0.087	0.056	0.084	0.081	0.194	0.074	0.091				
REL	0.184	0.057	0.044	0.423	0.088	0.076	0.037	0.042	0.067	0.231			
T	0.064	0.148	0.132	0.274	0.042	0.129	0.098	0.112	0.049	0.073	0.143		
V	0.128	0.040	0.052	0.542	0.042	0.080	0.031	0.093	0.055	0.180	0.540	0.070	

**Source(s):** Authors’ own work

**Table 4.** Discriminant validity assessment (Fornell−Larcker criterion)

	AS	AB	D	EE	GWB	IAC	MDA	PLV	RD	RES	REL	T	V
AS	0.821												
AB	0.114	0.855											
D	−0.002	−0.231	0.793										
EE	−0.130	−0.226	0.146	0.842									
GWB	−0.053	−0.198	0.214	0.100	0.812								
IAC	0.054	0.202	−0.163	−0.208	−0.175	0.862							
MDA	−0.012	−0.057	0.050	0.164	0.090	−0.004	0.809						
PLV	0.039	−0.125	0.188	0.195	0.398	−0.136	0.156	0.832					
RD	0.057	0.085	0.197	−0.017	0.110	−0.071	−0.107	0.038	0.828				
RES	0.079	−0.022	0.038	0.012	−0.006	−0.028	0.160	0.014	0.015	0.796			
REL	−0.178	0.044	−0.004	0.365	−0.061	0.044	0.024	−0.017	0.034	−0.196	0.840		
T	−0.012	−0.122	0.096	0.241	0.014	−0.112	0.081	0.090	−0.020	0.020	0.126	0.855	
V	−0.077	−0.014	0.009	0.460	−0.015	−0.062	0.020	0.058	−0.049	−0.145	0.460	0.058	0.868

**Source(s):** Authors’ own work

**Table 5.** Goodness-of-fit test

Metric	EME	SVQ	ADL → EME	ADL → SVQ	EME → SVQ
*R*²	0.062 (*p* = 0.021)	0.226 (*p* = 0.000)			
*f*²			0.066 (*p* = 0.035)	0.000 (*p* = 1.000)	0.273 (*p* = 0.000)
*Q*²	0.056	0.008			
RMSE	0.977	1.008			
MAE	0.794	0.750			

**Source(s):** Authors’ own work

### 4.2 Structural model assessment

In this study, four hypotheses were tested after validating the measurement model, and the structural model was assessed using the bootstrapping approach with 5,000 resamples in SmartPLS 4, as recommended by Hair *et al.* (2017). In Table 6, the goodness-of-fit test results demonstrate the model’s explanatory power and predictive relevance. The *R*^2^ results indicate that adaptive leadership accounts for 6.2% of the variance in employee engagement with a significance level of *p* = 0.021 and 22.6% of the variance in service quality with *p* = 0.000, suggesting a moderate effect, as per Cohen (1988). The *f*^2^ effect size shows that adaptive leadership has a small but significant impact on employee engagement at 0.066 (*p* = 0.035), while its effect on service quality is negligible at 0.000 (*p* = 1.000). Conversely, employee engagement exhibits a substantial impact on service quality with an *f*^2^ value of 0.273 (*p* = 0.000). The predictive relevance (*Q*^2^) values are 0.056 for employee engagement and 0.008 for service quality, indicating limited predictive accuracy (Hair *et al.*, 2019). Furthermore, the RMSE and MAE values for both constructs are close to 1, suggesting an acceptable model fit. Overall, these findings emphasize the crucial mediating role of employee engagement in the relationship between adaptive leadership and service quality.

**Table 6.** Summary of hypothesis testing

Hypothesis	Path	Original sample (β)	*T*-statistics	*P*-values	Decision
*H1*	ADL → SVQ	0.032	0.543	0.587	Not supported
*H2*	ADL → EME	0.302	5.411	0.000	Supported
*H3*	EME → SVQ	0.328	6.745	0.000	Supported
Indirect effect (mediation)					
*H4*	ADL → EME → SVQ	0.099	4.239	0.000	Supported

**Source(s):** Authors’ own work

In Table 6, the results of the hypothesis testing based on the PLS-SEM analysis show varying levels of support for the proposed relationships. Specifically, the direct effect of adaptive leadership (ADL) on service quality (SVQ) (*H1*) was not supported, as indicated by a nonsignificant path coefficient (β = 0.032), a low *t*-statistic (0.543) and a *p*-value of 0.587. In contrast, the direct effect of ADL on employee engagement (EME) (*H2*) was supported, with a significant path coefficient (β = 0.302), a high *t*-statistic (5.411) and a *p*-value of 0.000. Similarly, the direct effect of EME on SVQ (*H3*) was also supported, showing a strong relationship (β = 0.328), a high *t*-statistic (6.745) and a *p*-value of 0.000. Furthermore, the indirect effect (mediation) of ADL through EME on SVQ (*H4*) was significant, with a positive path coefficient (β = 0.099), a *t*-statistic of 4.239 and a *p*-value of 0.000, indicating full mediation.

## 5. Discussion

The primary objective of this study was to examine the intricate relationships between adaptive leadership (ADL), employee engagement (EME) and service quality (SVQ) within the unique context of Ethiopian private health-care settings. Our analysis, using PLS-SEM, yielded several key findings that both corroborate and diverge from existing literature, offering novel contributions to the field.

The statistically insignificant direct relationship between adaptive leadership (ADL) and service quality (SVQ) (*H1*) suggests that the benefits of adaptive leadership on service quality may not be immediately apparent or directly translated without the influence of intervening factors. This finding contrasts with some prior studies (e.g. Rojko *et al.*, 2025) that have suggested a more direct positive link. However, our results highlight the potential complexity of this relationship in the health-care context, where frontline employee behaviors and attitudes may act as crucial conduits for leadership effectiveness to manifest in improved patient experiences.

Conversely, the strong positive direct effect of adaptive leadership (ADL) on employee engagement (EME) (*H2*) aligns robustly with existing theoretical frameworks and empirical evidence (Curado and Santos, 2022; Uhl-Bien and Arena, 2018). Adaptive leaders, by empowering employees, fostering collaboration and encouraging a learning-oriented environment, appear to cultivate a more engaged workforce in Ethiopian private hospitals. This underscores the universal principle that leadership behaviors that value employee input and development contribute significantly to their motivation and commitment.

The significant positive direct effect of employee engagement (EME) on service quality (SVQ) (*H3*) strongly supports the well-established notion that engaged employees are more likely to deliver superior service (Bakker and Albrecht, 2018; Boyle *et al.*, 2023). In the demanding health-care environment, engaged employees are likely to exhibit greater empathy, proactivity and a stronger commitment to patient care, directly translating into positive service quality outcomes. This finding reinforces the critical role of a motivated workforce in achieving organizational goals related to patient satisfaction and care excellence.

The most compelling finding of this study is the significant indirect effect of adaptive leadership (ADL) on service quality (SVQ) through employee engagement (EME) (*H4*). This mediation effect clarifies the pathway through which adaptive leadership influences service quality. While ADL may not directly impact SVQ, its positive influence on employee engagement creates a more motivated and committed workforce, which in turn drives improvements in service quality. This nuanced understanding extends beyond simple direct relationships and highlights the crucial role of employee engagement as a mechanism for translating leadership effectiveness into tangible service outcomes in the Ethiopian health-care context.

Our finding regarding the indirect effect of adaptive leadership on service quality through employee engagement offers a more granular understanding compared to studies that have primarily focused on direct relationships. While some research such as MS *et al.* (2023) has linked adaptive leadership to improved service quality indirectly through engagement, our study provides empirical evidence of this specific mediating mechanism within the under-researched context of Ethiopian private hospitals. This context is particularly important as leadership dynamics and employee responses may be influenced by cultural and socio-economic factors that differ from Western settings where much of the existing leadership research originates.

Furthermore, our study contributes to the ongoing discussion about the relative importance of different leadership approaches in the digital age. While digital leadership is gaining prominence (AlNuaimi *et al.*, 2022; Malik *et al.*, 2024), our findings underscore the enduring importance of adaptive leadership principles in fostering the human capital (engaged employees) necessary for delivering high-quality care. This suggests that even as digital transformation progresses in health care, leadership approaches that focus on empowering and motivating the workforce remain fundamental to achieving service excellence.

This study offers a novel contribution through its contextual focus on the Ethiopian private health-care sector, its empirical validation of employee engagement as a mediator between adaptive leadership and service quality, and its advancement of understanding regarding leadership influences on service outcomes in a Sub-Saharan African country context.

## 6. Conclusion

This study offers important empirical insights into the complex interplay between adaptive leadership, employee engagement and service quality within Ethiopian private hospitals. The results demonstrate that adaptive leadership, characterized by flexibility, responsiveness and support for employee autonomy, does not directly result in immediate improvements in service quality. Instead, its influence is realized through its significant positive effect on employee engagement, a critical mediating mechanism. Engaged employees, who exhibit higher levels of commitment, enthusiasm and discretionary effort, serve as the vital conduit through which adaptive leadership translates into superior service outcomes. This finding underscores that leadership interventions aimed solely at operational targets, without addressing employee engagement, may be insufficient to drive sustainable improvements in service quality.

Moreover, the study highlights the broader importance of context in leadership research, emphasizing that strategies effective in Western settings may manifest differently in Sub-Saharan African health-care environments, where organizational structures, resource constraints and cultural dynamics may influence leadership effectiveness.

For health-care organizations operating in similar settings, these findings suggest that leadership development programs should not only focus on building adaptive capacities in leaders but also embed employee engagement as a strategic organizational priority. Future interventions should incorporate mechanisms for empowering employees, recognizing contributions and fostering a culture of continuous learning and adaptation. Ultimately, by cultivating adaptive leadership practices that actively promote workforce engagement, health-care institutions can create resilient, patient-centered service systems capable of delivering sustained quality improvements even amid external challenges.

## 7. Implications

### 7.1 Theoretical implications

The study provides several important theoretical contributions to the literature on leadership and organizational behavior. First, it advances the theory of adaptive leadership by empirically demonstrating its indirect impact on the quality of services through the facilitating role of employee involvement. Adaptive leadership is often theorized to be effective in complex environments, but little empirical work has been done to explore the mechanisms by which it drives results, especially in resource-limited and institutionally different contexts. By focusing on the Sub-Saharan African health sector, the study provides a more nuanced understanding of adaptive leadership outside developed country environments, where hierarchical cultures and scarce resources can change the dynamics of leadership.

Second, the study extends the JD-R model by identifying adaptive leadership as a key job resource that enhances employee engagement and, in turn, service quality. This supports the premise that leadership can mitigate job demands in high-pressure work environments, such as health care, by fostering engagement as a motivational pathway to improved performance outcomes.

Third, by generating empirical insights from a developing country context, the study contributes to ongoing efforts to globalize leadership theory. It underscores that while certain leadership principles may be broadly relevant, their expression and effectiveness are shaped by local cultural and institutional realities. These findings call for more context-sensitive theory building and validation across diverse settings. Moreover, the study deepens our understanding of the complex interaction between adaptive leadership, engagement and service quality, and invites further research into how leadership theories manifest and function across varying organizational ecosystems.

### 7.2 Managerial implications

The findings of this study present several practical and actionable implications for managers and administrators in Ethiopian private hospitals, and potentially for health-care institutions in similar developing country contexts. First, health-care organizations should prioritize the development of adaptive leadership at all managerial levels. This involves implementing targeted training programs that equip leaders with the skills to diagnose adaptive challenges, foster psychological safety, empower employees and facilitate collaborative problem-solving. Second, employee engagement should be treated as a strategic priority, given its critical role in linking leadership practices to service quality. Management should adopt strategies to boost motivation, commitment and participation among staff by providing opportunities for professional development, recognizing contributions, promoting an inclusive work environment and maintaining open communication. Third, empowering frontline staff is essential. Adaptive leadership promotes the active involvement of employees in decision-making and encourages innovative, ownership-driven approaches to patient care. Fourth, hospitals should institutionalize regular assessment and monitoring of employee engagement through surveys and feedback mechanisms to identify areas for improvement and sustain workforce morale. Finally, leaders must recognize that their influence on service quality often occurs indirectly through the engagement and empowerment of their teams rather than solely through direct interventions. A leadership approach that nurtures commitment and autonomy is more likely to yield sustained improvements in service delivery.

## 8. Limitations

Although this study has made contributions, there are several limitations that should be carefully considered. First, it is challenging to determine the temporal precedence of adaptive leadership, employee engagement and service quality due to the limitations of cross-sectional research design, which limits causal inference. Future studies that use experimental or longitudinal designs may be better able to depict how these relationships are dynamic and change over time.

Second, the study is geographically and contextually bounded, focusing exclusively on private hospitals in Addis Ababa, Ethiopia. While this focus enhances contextual depth, it limits the generalizability of findings to public health-care institutions, rural areas, other sectors or different cultural settings. Given the distinct institutional and organizational characteristics of health-care systems across regions, future studies should replicate and extend this work in more diverse environments to assess the robustness and transferability of the proposed model.

Third, the reliance on self-reported data for key constructs such as employee engagement and leadership behaviors introduce the possibility of common method variance. Although we used procedural remedies and statistical controls, future research could benefit from triangulating data sources, for example, incorporating patient satisfaction metrics or 360-degree leadership assessments to mitigate this bias and enrich construct validity.

Finally, while efforts were made to control for key confounding variables, unobserved factors such as organizational climate, leadership tenure or team composition may have influenced the observed relationships. Future studies should consider these additional variables to further isolate the unique effects of adaptive leadership on performance outcomes.

## 9. Future directions

Future research should build upon these findings in several ways. Longitudinal studies are needed to establish the causal direction and long-term impact of adaptive leadership on employee engagement and service quality. Investigating the moderating effects of organizational culture, job satisfaction or specific contextual factors considering hospital size, specialization could provide a more nuanced understanding of these relationships. Comparative studies across different sectors within Ethiopia and in other developing nations would enhance the generalizability of the findings. Using mixed approaches, combining quantitative data with qualitative insights from interviews and focus groups, could provide richer explanations of the underlying mechanisms. Finally, exploring the role of other leadership styles in conjunction with adaptive leadership could offer a more comprehensive understanding of effective leadership strategies in health care.
